# Observing vegetation phenology through social media

**DOI:** 10.1371/journal.pone.0197325

**Published:** 2018-05-10

**Authors:** Sam J. Silva, Lindsay K. Barbieri, Andrea K. Thomer

**Affiliations:** 1 Department of Civil and Environmental Engineering, Massachusetts Institute of Technology, Cambridge, Massachusetts, United States of America; 2 Rubenstein School of Environment and Natural Resources and Gund Institute for Environment, University of Vermont, Burlington, Vermont, United States of America; 3 School of Information, University of Michigan, Ann Arbor, Michigan, United States of America; University of Waterloo, CANADA

## Abstract

The widespread use of social media has created a valuable but underused source of data for the environmental sciences. We demonstrate the potential for images posted to the website Twitter to capture variability in vegetation phenology across United States National Parks. We process a subset of images posted to Twitter within eight U.S. National Parks, with the aim of understanding the amount of green vegetation in each image. Analysis of the relative greenness of the images show statistically significant seasonal cycles across most National Parks at the 95% confidence level, consistent with springtime green-up and fall senescence. Additionally, these social media-derived greenness indices correlate with monthly mean satellite NDVI (r = 0.62), reinforcing the potential value these data could provide in constraining models and observing regions with limited high quality scientific monitoring.

## Introduction

Vegetation phenology is an important control on the global fluxes of energy, water, and carbon from terrestrial ecosystems [1], and is a useful indicator of ecosystem response to climate change [2]. It is therefore important to understand and monitor vegetation phenology across the globe. Advances in satellite observational techniques have allowed for monitoring of phenological characteristics at the global to regional scales [3]. At more local scales, scientific imagery collection networks such as Phenocam have provided high-resolution data that have substantially improved our understanding of phenological response to changes in environmental and climatic conditions [4]. Here we explore the value of an additional source of data for the study of vegetation phenology: images posted to the social media website Twitter.

In recent years, online social media platforms have become an increasingly important source of information regarding real time events across the globe. With more than two thirds of online American adults as active users [5], immense quantities of images, text, and other data are posted to these social media webpages daily: Facebook users, for instance, post more than 600 TB of data per day [6], and Twitter users posted an estimated 600 tweets per second in 2010 [7], though some argue that number has subsequently fallen [8]. These data have been shown to be useful in scientific studies in a range of fields, including monitoring clusters of notable events [9], tracking trends in regional influenza outbreaks [10], and investigating the value that these data can provide to the earth and environmental sciences [11]. Case et al. [12] successfully use data from Twitter to track and map auroral activity, and improve forecasts of when and where aurora are likely to occur. Ford et al. [13] use data from Facebook to estimate wildfire smoke exposure, and find that adding information from Facebook leads to an improved exposure assessment. Additionally, the United States Geological Survey has developed methodology to use messages posted to Twitter to supplement earthquake detection networks [14].

Many of the images posted to these social media pages are of the natural world, or contain the natural world within their field of view. For example, there are many images of U.S. National Parks posted to social media webpages daily by park visitors, ranging from direct photographs of large scale natural panoramas to the park existing as the backdrop to family pictures or self-portraiture (“selfies”). This collection of images is in contrast to many large scale monitoring citizen science networks (e.g. [15]) because this data is not directed at any specific scientific objective. In this way, these posted images could serve as “ambient data”, capturing the state of the natural world without any prescribed scientific purpose.

Direct digital photography of vegetation has been shown to be a valuable technique for the investigation and understanding of plant phenology [16]. A common approach is to use color indices derived from the digital imagery to quantify the change in vegetation color with time, which can then be related to a change in phenology. In a similar vein, we hypothesize that with proper selection criteria and a large enough sample size, the phenological variability of vegetation should be represented in this ambient data posted to social media. Individual personal cameras are likely not useful for the image processing typically associated with phenological scientific analyses. This is due to the fact that the calibration and sensitivity of an individual personal camera is not known, unlike modern phonological work using digital cameras (e.g. [16]). However, acquired en masse, this data could potentially provide useful information. Similar work using images submitted to the image sharing website Flickr (www.flickr.com) to observe wildflower phenology in Mount Rainier National Park, found results from the social media page were largely consistent with observations by professional scientists [17].

In this work, we investigate the information regarding local vegetation phenology contained within de-identified aggregated images posted on Twitter. We compare these results to satellite observations of plant phenology, and describe the potential value and shortcomings in using social media as a source of data in the earth and environmental sciences.

## Data and methods

### Twitter images

**Fig 1:**
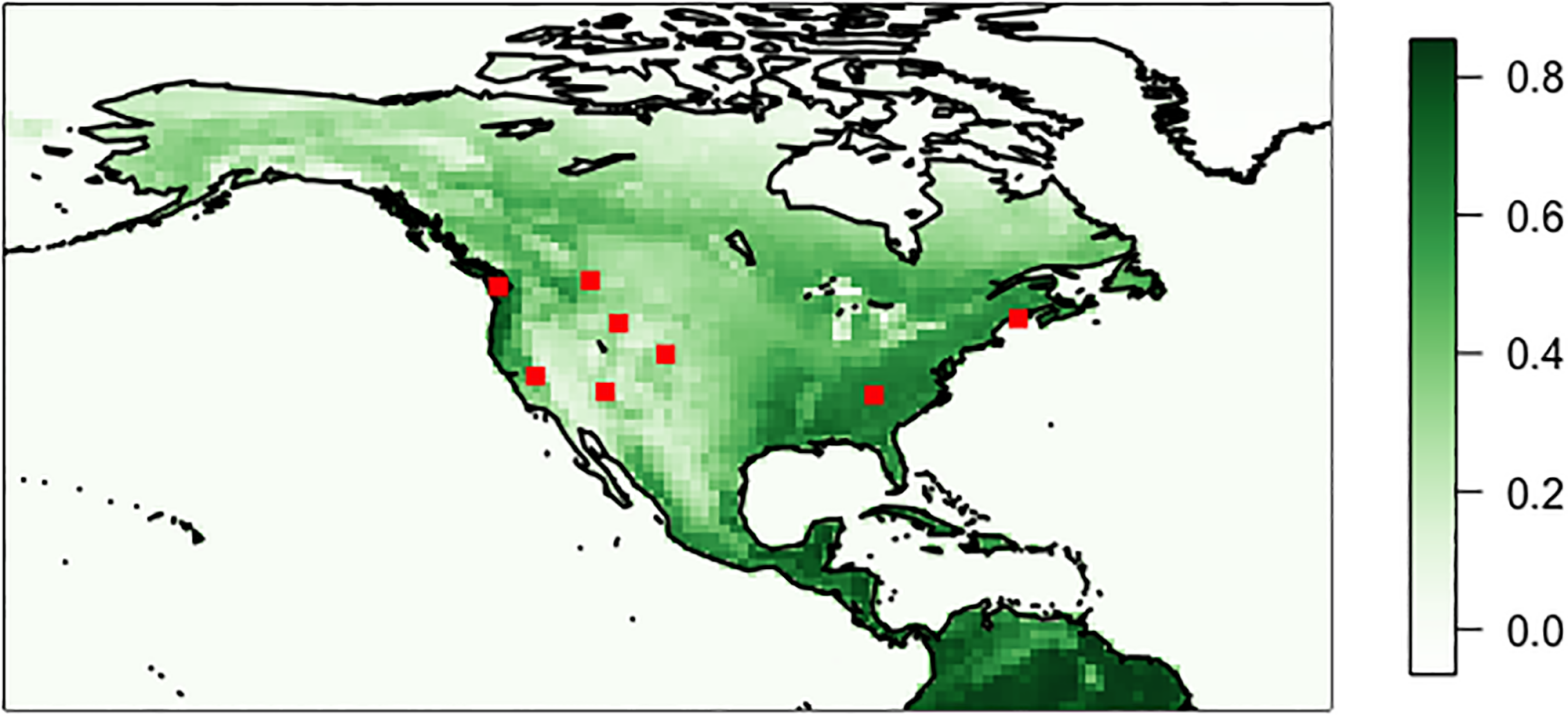
Annual average MODIS Terra NDVI. Red points represent the locations of the selected national parks.

For this investigation, we consider images posted within eight of the most frequently visited U.S. National Parks (see Table 1). National Parks are regions where images are frequently posted to Twitter, and those images commonly contain the natural world within their field of view. Selection of images taken at these parks allows for the best opportunity to capture potential phenology within these ambient data and provide a range of geographic locations and physical and ecological features. The locations of these parks are shown as red points on Fig 1.

**Table 1:** The list of National Parks considered in this analysis, and the full name used as the search term for initial spatiotemporal filtering.

National Park Name
Acadia National Park
Glacier National Park
Grand Canyon National Park
Grand Teton National Park
Olympic National Park
Rocky Mountain National Park
Great Smoky Mountains National Park
Yosemite National Park

Images from Twitter were gathered using a combination of the Twitter web page and the freely available Twitter API (https://developer.twitter.com/). All tweeted images containing text with the full names of the national parks listed in Table 1 were downloaded by December 2016, from the years 2011 through 2015. We note that these freely available data collection techniques likely only sample a small fraction of all available and relevant information [18], however this still leads to a large total number of images available for analysis. The text-based search for each National Park was done instead of a geolocated or a regional search primarily due to a lack of geolocation information attached to most tweets and the fact that many of these images are potentially uploaded away from the exact location where they were taken (related to issues with cell phone service, internet access, etc.).

### Image analysis

Once downloaded, we removed all duplicate images and images smaller than 10kB. This filtering removed ~3% of the data, and was done to limit the influence of off-topic advertisements and other types of spam posted to Twitter, as well as remove very low resolution or poor quality images. These images were all downloaded as JPG files, with the common 3 dimensional color space of Red (R), Blue (B), and Green (G). From these three color channels, we calculated a mean greenness index per image, similar to [16]:

Greennessindex=G/(R+B+G)

This index is the ratio of the green channel to the sum of all channels within a given pixel, taken as a mean across the entire image. The greenness index as calculated here is a frequently used metric, and varies in space and time in a way that has been shown to characterize changes in plant phenology well [19, 20].

No additional filtering or image processing was applied, which resulted in the dataset being rather noisy (the monthly coefficient of variation is generally on the order of 0.1). We limited our filtering to what is presented here to keep this analysis tractable, as well as to take advantage of most of the data available. We note that more advanced image processing techniques, such as the machine learning approach in [21], would likely reduce the noise, however these are challenging to apply consistently across all parks.

**Fig 2:**
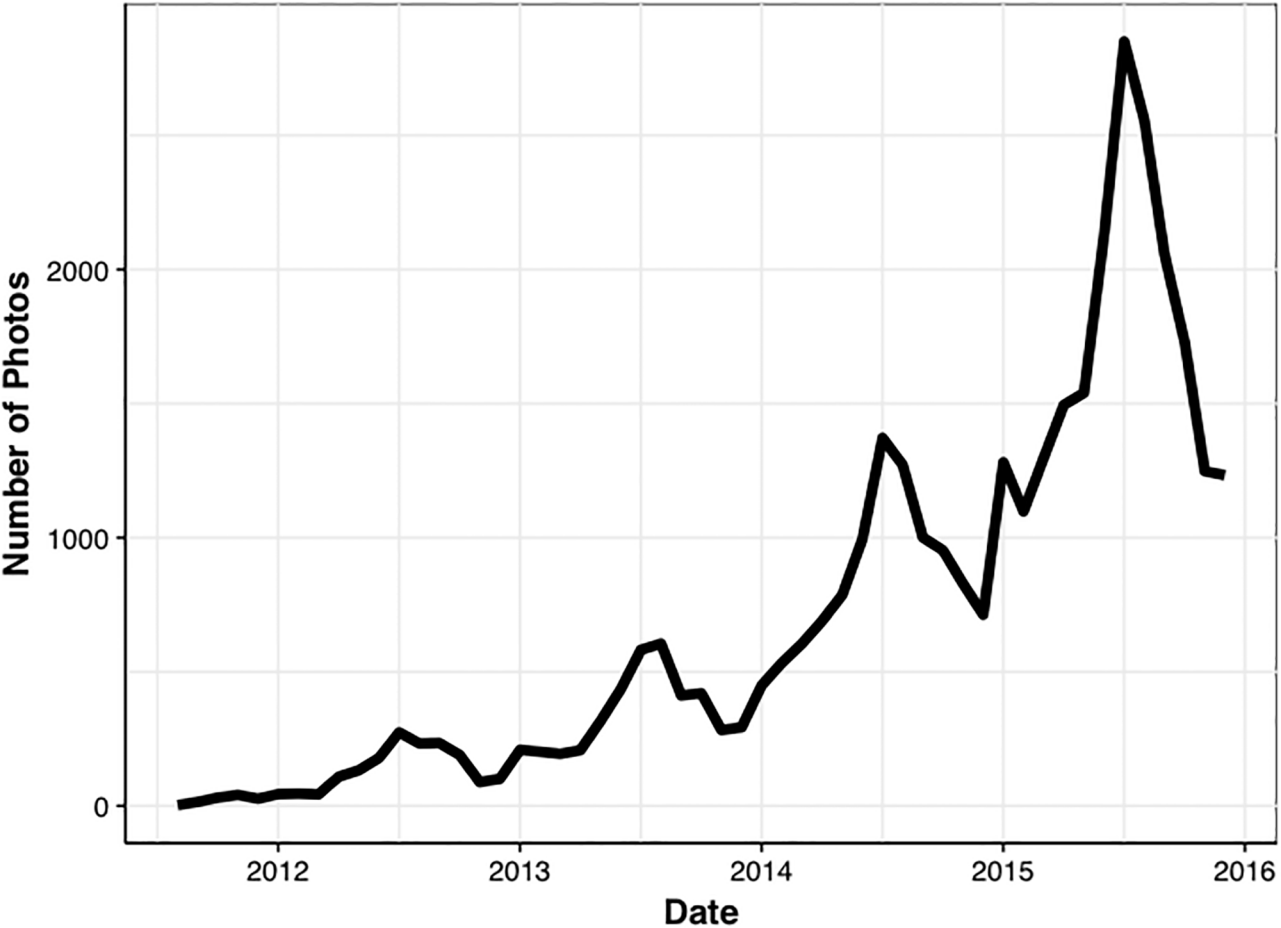
Number of images per month across all national parks, note the seasonal peaks in summertime.

To treat the noise in the data, we show results aggregated on relatively long timescales (seasonally and monthly) from here onward. Confidence intervals were estimated through a bootstrapping approach, where a given parameter (e.g. monthly mean greenness index, correlation, etc.) was computed 10,000 times on the data resampled to the length of the original dataset with replacement. We use only years 2014 and 2015 for this work due to the substantial increase in available images posted to Twitter after the year 2013, the total number of images per month are shown in Fig 2.

### Satellite data

We use the Normalized Difference Vegetation Index (NDVI) from the MODIS instrument onboard the NASA Terra satellite [22] to assess the results of the Twitter analysis. NDVI data have been used extensively in previous studies to directly constrain plant phenology as in [2], [3], and [23]. The wide spatial coverage of satellite observations enables comparison of the Twitter analysis across all parks with a single consistent instrument. Despite the fact that NDVI is calculated using different spectral channels (Near Infrared and Red, instead of digital RGB colorspace), NDVI observations have previously been shown to be useful to assess phenology derived through digital photography (as in [24]). Annual average MODIS NDVI over North America is shown in Fig 1.

Direct comparison of the satellite NDVI with the images from Twitter is not possible, because the exact region contained in the field of view of a given Twitter image is not known at the time of upload. To account for this, we used all satellite NDVI within 0.5° of the center of a park, aggregated on a monthly scale. These were then compared with the monthly averaged greenness indices for every park, and a bootstrapped correlation was calculated, following the bootstrapping methodology described above.

## Results and discussion

### Seasonal analysis

**Fig 3:**
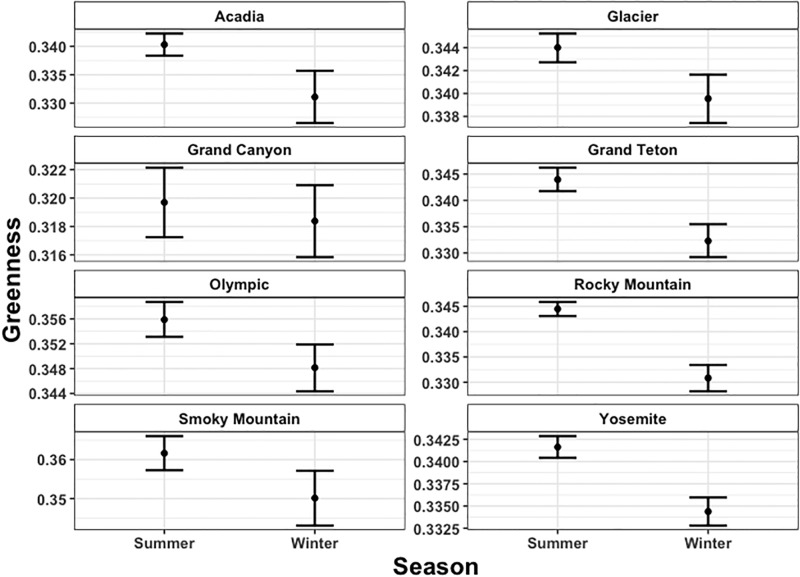
Seasonal greenness index across National Parks. The bars represent the 95% Confidence Interval calculated through a bootstraping approach.

As an initial test of the fidelity of the Twitter observations, we compared computed average greenness indices for all images uploaded during summer (June, July, and August) with winter (December, January and February) for the 8 National Parks selected in this work. These seasonal comparisons are summarized in Fig 3. From the mean values and confidence intervals in Fig 3, it is evident that for 7 of the 8 parks, the summer average greenness index is higher than during the winter, at the 95% confidence interval. The summer average greenness index over the forested national parks was generally ~0.34, while the winter average was usually lower by ~0.05 to 0.1. The highest seasonal greenness indices were from images taken of Smoky Mountain National Park, at greater than 0.36, and the lowest indices were of the Grand Canyon National Park, at less than 0.32. These results are consistent with the hypothesis that these data reflect the dynamics of the regional plant phenology. The only park that does not follow this seasonal pattern in significance is the Grand Canyon, where the summer index is higher than the winter index, though not significant at the 95% confidence interval. This is potentially due to the relatively small variability in the vegetation cover across the Grand Canyon National Park, as well as sampling bias; visitors attending and photographing the Grand Canyon are commonly focused on capturing images of various geological formations rather than vegetation.

### Monthly analysis

**Fig 4:**
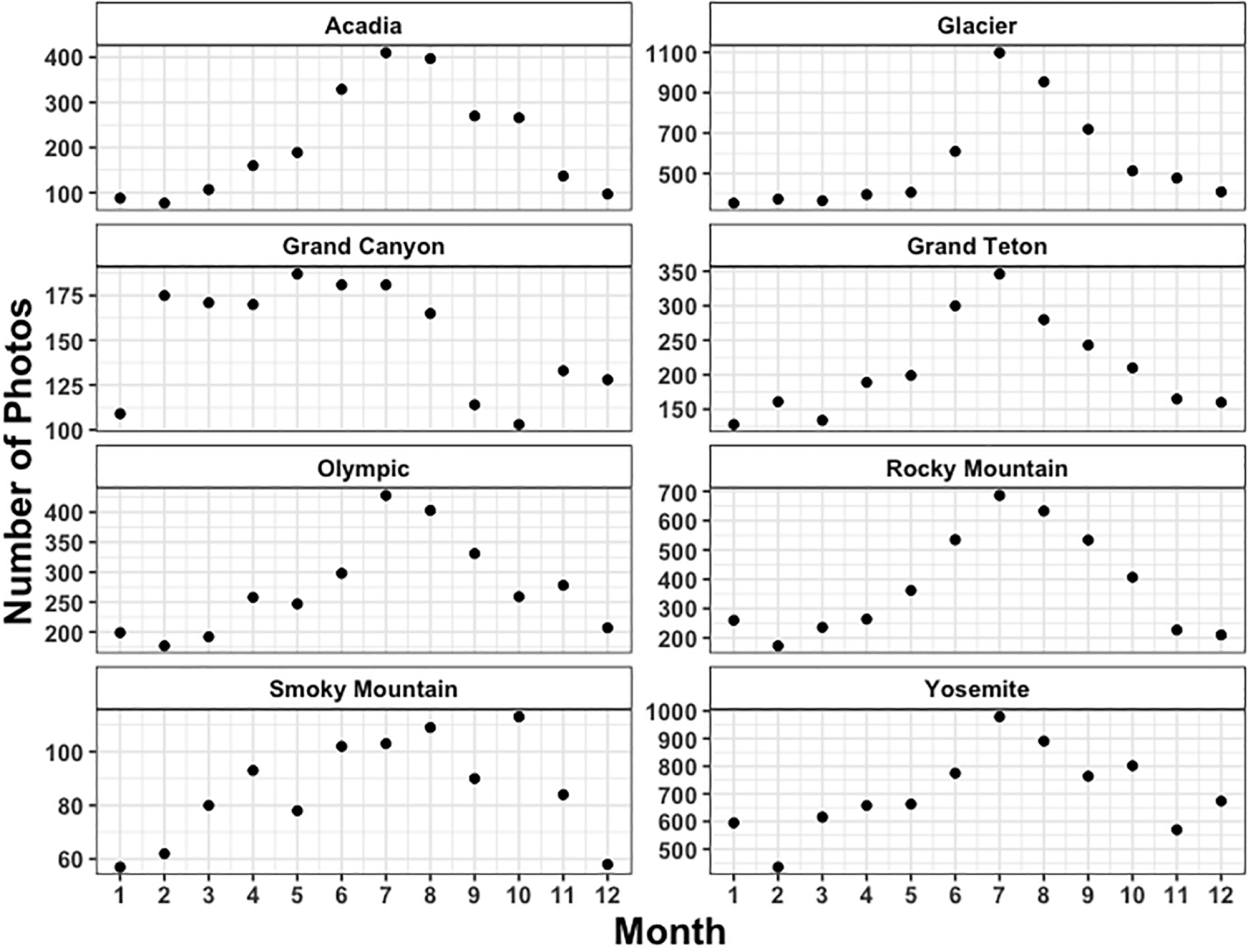
Monthly number of observations after filtering (removal of duplicates and pictures smaller than 10kB).

Given the positive results of the seasonal analysis, we increased the time resolution of the analysis to investigate the variability in monthly average greenness indices. The total number of images posted per month is shown in Fig 4. It is apparent from both Figs 2 and 4 that there is generally a seasonal cycle in the posting of National Park images to Twitter, which is consistent with summer travel to these locations. The summer months contain the most images, and the winter is generally the least photographed. The number of images posted per month ranges from less than 100 in the winter months for the Smoky Mountain National Park, to more than 1000 in July for Glacier National Park. Given that the greenness index reported here is calculated as an average of the total number of images posted, the variability in the number of observations should not directly influence our reported indices. However, the variability will potentially increase the range of the confidence intervals on those monthly means with fewer observations. An interesting feature in the monthly number of observations is the relative peak in number of images posted in October for Smoky Mountain National Park. These images frequently contain the colors of fall foliage and are consistent with a peak of so-called “leaf peepers”, individuals who travel to the parks for the specific reason of seeing this fall foliage.

**Fig 5:**
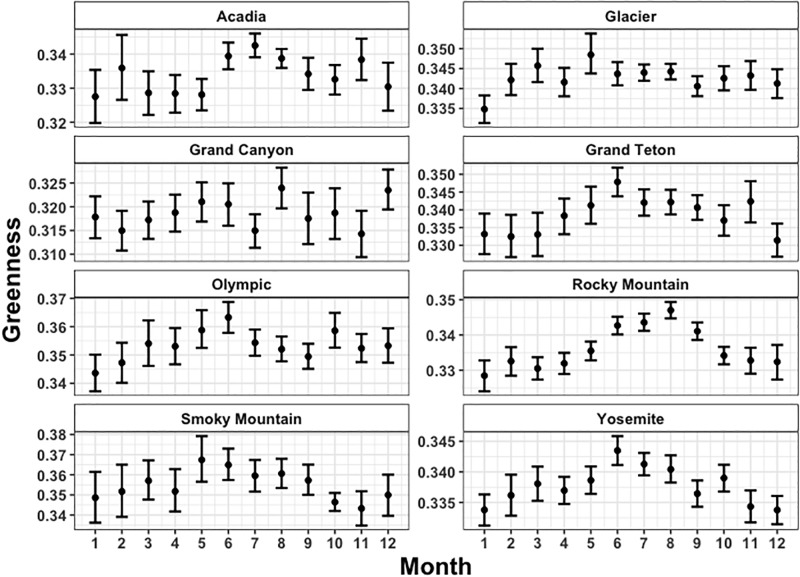
Monthly greenness index across National Parks. The bars represent the 95% Confidence Interval calculated through a bootstrap approach.

The monthly Twitter-observed greenness indices across all national parks are shown in Fig 5, along with bootstrapped 95% confidence intervals. Maximum mean greenness indices are ~0.37, and the lowest are below 0.32. In general, values in the summer months are higher than those in winter months. However, the statistical significance of the higher summer months is challenged by the wide confidence intervals from months with few observations, which frequently occur during winter. For all parks the highest month is higher than the lowest month, at the 95% confidence level. That highest month always occurs in the summer months (May-July), and the lowest month during the winter (November—February). As evident in Fig 5, the Rocky Mountain National Park images have the strongest seasonal cycle, with a sharp rise in the spring months, and sharp dropoff in autumn. The Grand Canyon National Park has the least seasonality of all the parks, with no real seasonal signature at all. Similarly to the seasonal-scale analysis, we attribute this lack of a seasonal cycle over the Grand Canyon National Park to a relatively small seasonal change in vegetation in the desert region, and sampling bias away from vegetation. Glacier National Park also has fairly low seasonal variability, despite a large swing in actual vegetation phenology throughout the year. This is due to a multitude of reasons, including the fact that many of the downloaded images of Glacier National Park contain substantially more advertisements of various travel and outdoor recreational products. These advertisements frequently include an image of Glacier National Park during the summer even when posted during non-summer months, which spuriously increases the mean greenness index. In general, the other 5 national parks show a reasonably strong seasonal cycle, with one or two months throughout the year appearing outside of the seasonal pattern. These results demonstrate the challenges associated with the large amounts of noise that exist in data obtained through social media.

### Comparison with satellite NDVI

**Fig 6:**
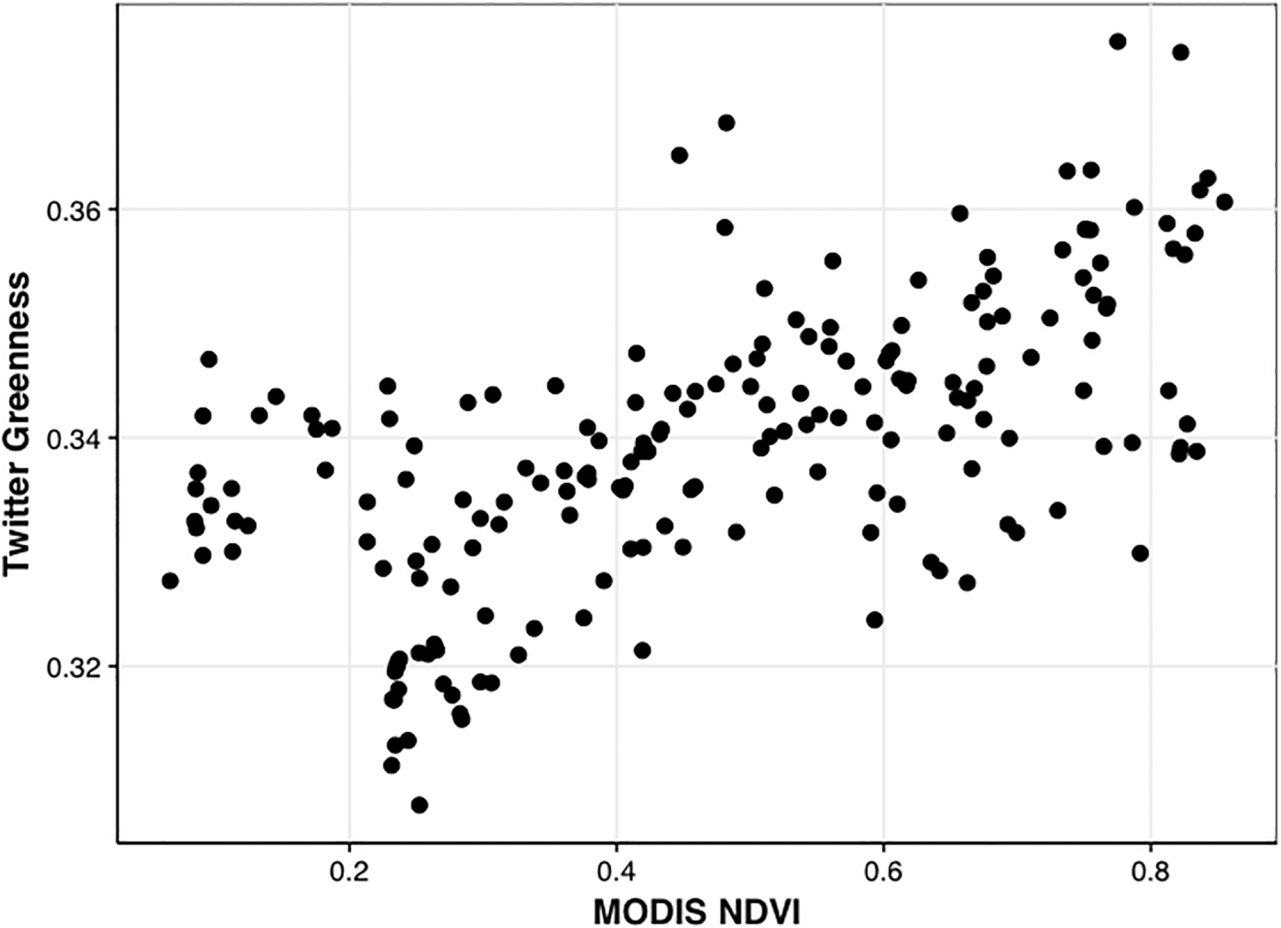
Monthly Twitter-derived average greenness index vs. MODIS NDVI for all parks. Pearson’s correlation coefficient is ~0.62.

Satellite observations of NDVI are used as an independent source of data to assess the analysis presented in this work. Monthly mean NDVI pixels were selected over each national park, and compared with the Twitter analysis on a monthly per park basis. This comparison is shown in Fig 6. Satellite NDVI and the Twitter greenness index are positively correlated, with a Pearson’s correlation coefficient of 0.62 (95% CI 0.54 to 0.70). The spearman’s rank correlation is nearly identical (0.63). The slope of this line is not expected to be near 1, since the greenness index and NDVI are fundamentally different quantities. This positive correlation with NDVI is also found in other studies, including [24], who found a spatial correlation of 0.62 using scientific quality cameras to derive greenness indices at a single site. These significant positive correlations within the satellite observations keep with the original hypothesis that the phenological variability of vegetation should be represented in this ambient data. We do not show monthly correlations across individual parks because correlation coefficients are known to be unstable for small comparisons (12 points), and are thus not a useful metric here.

### Evaluation of Twitter images as a source of data

Though the positive correlation with NDVI is a promising indicator that the phonological dynamics within National Parks is represented in these data, we recognize that there are disadvantages present when analyzing a large volume of data from a social media source. Many of those disadvantages center around the fact that these individual images are not validated or calibrated in the same way as other high fidelity field observations [16]. This creates challenges in dealing with quality control filtering and proper spatial attribution of a given posted tweet.

The most direct quality control filtering challenge is the inclusion of some incorrect images, misidentified as containing a National Park. For example, some images not containing the outside world are still collected through our text-based search. This could be remedied using various machine learning approaches (e.g. [21]). Additional variability and uncertainty is introduced from changes in lighting conditions and user post-processing. The greenness index of an image of a leaf on a cloudy day can be different than an image in direct sunlight. Many of these quality control filtering and calibration issues are remedied in field sites through the use of a reference panel placed in the camera field of view, which of course is not available in this study of ambient data. Lastly, we make no attempt to correct for “filters” and other types of post-processing applied to images prior to their posting to Twitter.

**Fig 7:**
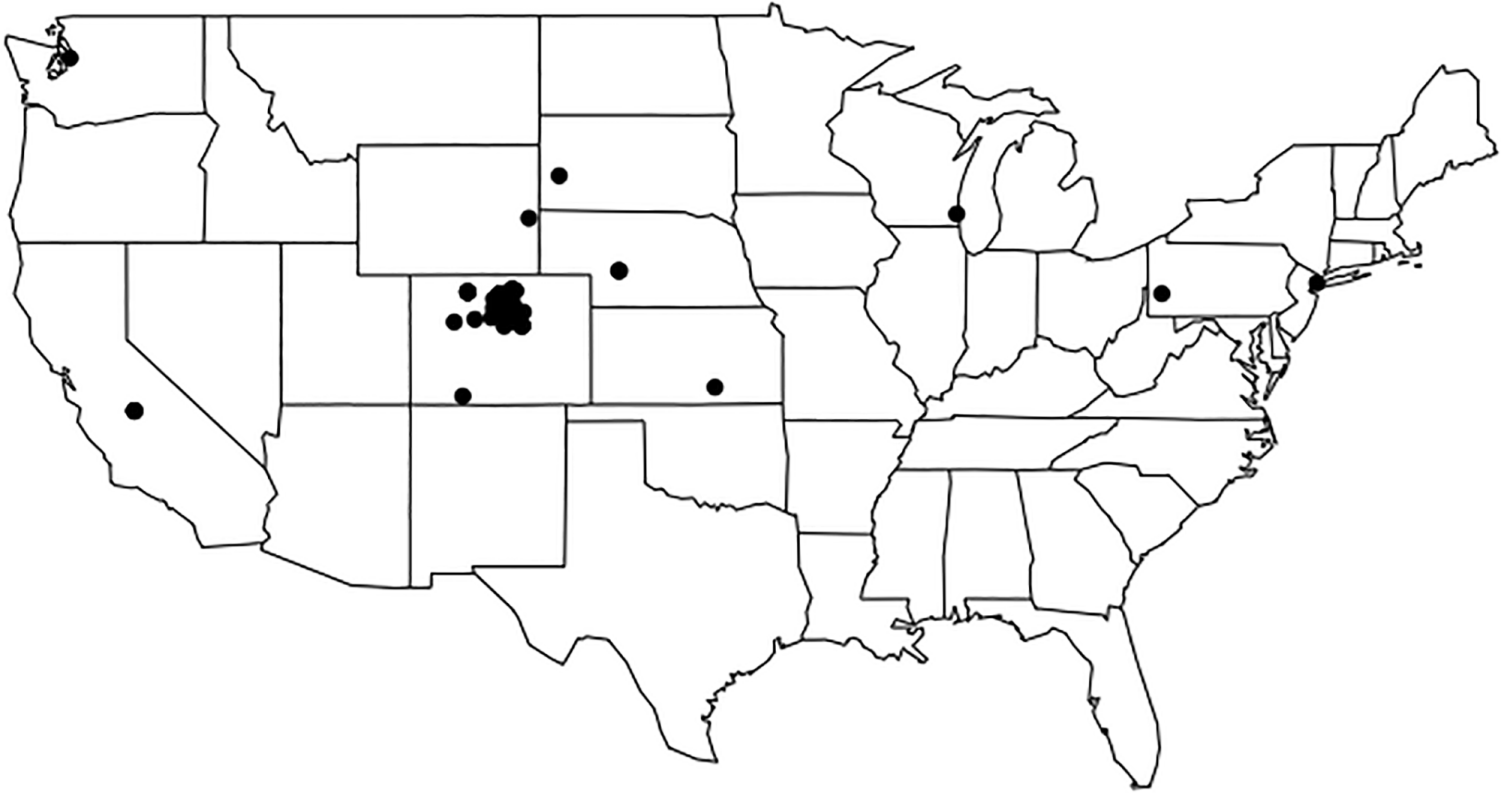
The spatial distribution of geotagged images from Rocky Mountain National Park used in this work. Two upload locations are masked in Europe, to better demonstrate the spatial variability across the United States.

We attempted to improve spatial attributions of Tweets by requiring the full name of a given park to be present in the text associated with an image. This likely reduced our total amount of available data substantially. This is demonstrated by the relatively small number of images posted over Smoky Mountain National Park, which is counterintuitive given that it is the most frequently visited park in the United States (https://irma.nps.gov/Stats/). This text search was done instead of a direct geolocation based search due to technical limitations using the free version of the Twitter API, where the number of available Tweets is “rate limited” to a certain number per hour, and free access does not allow for full-stream historical searches. We investigated the potential applicability of tweets that contain geolocation information using a limited set of data from this analysis, the Rocky Mountain National Park set of images. Only ~2% of all downloaded tweets from this work in this park were geotagged, with very poor temporal coverage. If we were to only use geotagged images, the monthly and seasonal variability analysis done here would be impossible due to a lack of data. However, the spatial coverage is quite promising, with nearly one third of all geotagged images within, or very near to, the park boundaries. Fig 7 shows the spatial distribution of the geolocated images matches the location of Rocky Mountain National Park well. Note that there are two points from geotagged uploads in Europe that are masked in Fig 7. As geolocation improves, and social media images can be more readily traced to the location where they were taken, this issue will become more resolved.

Our work shows that despite these disadvantages, these data reproduce the broad phenological characteristics of various ecosystems across the U.S. National Parks, consistent with satellite observations of NDVI and ground-based field studies. As in many other studies of ambient data from social media (e.g. [10,12]), we leverage the large amount of available data against the large inherent uncertainty of an individual posted image. Further work into the quality of images posted by specific users (e.g. amateur photographers vs family photographs) or using alternative social media platforms (e.g. Flickr or Instagram) would help constrain the sources of variability and potential uncertainties within these data.

Due to the stated challenges associated with these data, we of course do not suggest that they supersede high fidelity surface or space-based observations, such as those from the Phenocam sites. However, there are several substantial advantages to the use of these ambient data. The first is that there are vast amounts of this data currently available. For example, Facebook stores much more data per day (~600 TB/day, [6]) than NASA’s Earth Observing System Data and Information System (EOSDIS) data repositories (~6TB/day as of 2014, [25]). The fact that a small sample of all the images posted to social media as analyzed here have demonstrated potential value for scientific purposes indicates that it is likely that additional sources of ambient data from social media would be valuable for further study of vegetation phenology.

These ambient data are also vastly less expensive to collect than the higher quality field instruments. The data gathered through this work was all done on laptop computers using the freely available API from Twitter. The low cost of working with ambient data allows for much broader access to the data stream. Full access to all of the data from a given social media platform, the so-called “fire hose” of data, is far more expensive, and requires far more expertise, but allows for far more in depth analysis, and easier access to historical tweets. However, the technical and financial requirements for an analysis such as the one presented here are far lower.

An additional benefit of these data is that the spatial coverage of social media data outweighs that of dedicated field studies. The phenological dynamics of many regions that are poorly sampled by high fidelity observations could still be constrained through data similar to those presented in this work. Indeed, ambient social media data are already revolutionizing the data collection and analysis of recreation and tourism within National Parks and other natural landscapes [11, 26, 27, 28, 29]. Since these ambient social media data are transforming the ways visitors are being monitored, and are being used to support management decisions [29], the use of these social media data will likely expand in scale and scope to augment measures of visitation, and valuation for nature-based recreation and tourism.

While our study did capture general data trends that may be attributed to visitation (e.g. overall more images in the summer months, high number of images in autumn in parks notable for their fall foliage), we did not find evidence for correlation between the number of images and visitation rates or popularity, (e.g. Smoky Mountain National Park had the fewest images, despite it being the most visited park, https://irma.nps.gov/Stats/). Although there are challenges in using these ambient data for visitation [29], more refined methods of analysis such as counting the unique number of users posting images on a given date”photo-user-days” instead of total number of postings, and using data from a broader range of social media platforms including Flickr and Instagram, have been shown to correlate well with visitation [11, 26, 28] and for valuation of landscape and nature-based recreation ([27,28]). Understanding the full potential in the usage of these data, and the methods and analysis for both social and natural dynamics, is worth further consideration in future work.

Working with social media data does present some unique challenges, particularly in balancing the potentially conflicting needs to a) adhere to Twitter’s Terms of Service (TOS); b) use, share and store images in a way that would not unduly or unexpectedly impact the Twitter users who created and shared them; and c) still publish enough information about our dataset to make the project reproducible. Per the Twitter Terms of Service (TOS), we are only permitted to share and archive the unique identifiers of the tweets we used in our analysis, not the images themselves. This is just as well, given that the images may contain personally identifying information of their creators; while they have been made public, their sharers did not necessarily intend them to be used or made available as an aggregate (see [30] for further discussion of this issue). We consulted with our universities’ Institutional Review Boards (IRBs) to confirm that there were not additional steps that needed to be taken to ethical share our dataset. The IRBs stated that we did not need to apply for an exemption for oversight, given that we were not sharing the images themselves.

Our final dataset includes these unique IDs of each of the tweets containing images that we analyzed. This makes it possible for others to reproduce and verify our results using the Twitter API, while also protecting the identities of the Twitter users who shared their images, and adhering to Twitter’s TOS. We note, however, that recent changes to Twitter’s TOS have made some researchers and library and digital curation professionals concerned that this identity-obscuring, reproducibility-supporting feature may be in danger ([31]; later clarified by [32]). Twitter imposed limits on the number of Tweet IDs that may be published by non-academics as part of a dataset [33]; researchers affiliated with an academic institution have no such limits. Though these limits did not impact this project (both because of the authors’ affiliation with universities, and because of our dataset’s size) we think it is important to note that they could impact other projects. Continued research with Twitter data will be dependent on Twitter’s continued support of research use of Twitter data, which is by no means guaranteed.

## Conclusions

In this work we investigated the potential for images posted to the social media website Twitter to capture vegetation phenology in national parks. We found that these data reproduce the broad seasonal cycles consistent with the vegetation dynamics within given national parks, and that they exhibit a positive correlation with satellite observations of NDVI. With improved image processing techniques, as well as more available images, ambient data from social media could be used to constrain models and observe regions with limited high quality scientific monitoring.

## Supporting information

S1 Data The tweet IDs and associated National Parks used in this work. (CSV)
